# Photocrosslinked gelatin hydrogel improves wound healing and skin flap survival by the sustained release of basic fibroblast growth factor

**DOI:** 10.1038/s41598-021-02589-1

**Published:** 2021-11-29

**Authors:** Toshihiro Kushibiki, Yoshine Mayumi, Eiko Nakayama, Ryuichi Azuma, Kenichiro Ojima, Akio Horiguchi, Miya Ishihara

**Affiliations:** 1 Department of Medical Engineering, National Defense Medical College, 3-2 Namiki, Tokorozawa, Saitama Japan; 2 Department of Plastic Surgery, National Defense Medical College, 3-2 Namiki, Tokorozawa, Saitama Japan; 3 Department of Urology, National Defense Medical College, 3-2 Namiki, Tokorozawa, Saitama Japan

**Keywords:** Medical research, Biomaterials, Tissue engineering

## Abstract

Biomaterials traditionally used for wound healing can act as a temporary barrier to halt bleeding, prevent infection, and enhance regeneration. Hydrogels are among the best candidates for wound healing owing to their moisture retention and drug-releasing properties. Photo-polymerization using visible light irradiation is a promising method for hydrogel preparation since it can easily control spatiotemporal reaction kinetics and rapidly induce a single-step reaction under mild conditions. In this study, photocrosslinked gelatin hydrogels were imparted with properties namely fast wound adherence, strong wet tissue surface adhesion, greater biocompatibility, long-term bFGF release, and importantly, ease of use through the modification and combination of natural bio-macromolecules. The production of a gelatin hydrogel made of natural gelatin (which is superior to chemically modified gelatin), crosslinked by visible light, which is more desirable than UV light irradiation, will enable its prolonged application to uneven wound surfaces. This is due to its flexible shape, along with the administration of cell growth factors, such as bFGF, for tissue regeneration. Further, the sustained release of bFGF enhances wound healing and skin flap survival. The photocrosslinking gelatin hydrogel designed in this study is a potential candidate to enhance wound healing and better skin flap survival.

## Introduction

Wound healing is achieved through physiological events, such as inflammation, proliferation, and remodeling^1^, and is a matter of concern, particularly for post-surgery wounds, burns, and scars^2^. In addition, the incidence of diabetes mellitus (DM) continues to rise at an alarming rate. DM is associated with various complications, such as diabetic wounds, which affect 25% of patients with DM^3^. Therefore, new methods to improve the pathophysiology of diabetic wound healing remain warranted. Biomaterials could enhance wound healing^4,5^. Moreover, random cutaneous flaps are frequently used in reconstructive surgery due to its simplicity, flexibility, and easy-of-use. However, since these flaps do not have specific blood vessels, there remains a risk of ischemia and necrosis^6^. Previous studies have shown that accelerating the angiogenesis in flaps is beneficial to improve the survival of flaps^7,8^. Thus, biomaterials for enhancing the angiogenesis in cutaneous flaps are necessary^9^.

Biomaterials traditionally used for wound healing can act as a temporary barrier to halt bleeding, prevent infection, and enhance regeneration. Although several biomaterials, such as collagen and synthetic polymer, have been developed for wound-dressing, they remain unsuitable for clinical use^10,11^. Hydrogels are the best candidates for wound healing owing to their moisture retention and drug-releasing properties^12–14^. Hydrogels used in wet wound dressings are designed for wound cleansing, facilitating the removal of all necrotic, contaminated, or damaged tissue^15^. Recent progress in material sciences has facilitated the evaluation of chemical agents, such as fibrin glue, gelatin, collagen, and hydrogels, for rapid wound healing^16–18^. Historically, hydrogels were pre-formed and delivered to target sites in patients, however, uneven or irregularly shaped wound surfaces are difficult to cover entirely with pre-formed biomaterials, resulting in ineffective wound healing and low cell survival for tissue remodeling^19^. Hydrogel precursors can be injected with a syringe and can be locally cross-linked by percutaneous photopolymerization^20^. Injectable materials have several advantages over pre-formed materials^21^. A typical example is that they can be molded to the shape of the injection cavity, hence even non-standard shapes can be used immediately to help regenerate tissue^22^. Injectable hydrogels have suitable physicochemical properties for injection in situ into the body. Therefore, they have attracted interest as materials for drug delivery system and tissue engineering^23,24^. The cross-linking methods, based on chemical and physical linkages, are used to prepare hydrogels that are injectable^25,26^. These hydrogels exhibit relatively higher mechanical property and more suitable physicochemical properties ensuing greater durability over time due to stable covalent bonds^27^. However, these materials are inefficient owing to slow gelling, weak adhesion strength against wet tissue surface since their clinical applications are limited due to inflexibility or toxic degradation products^28,29^.

Photo-polymerization using visible light irradiation is a promising method for hydrogel preparation because it can easily control spatiotemporal reaction kinetics and rapidly induce a single-step reaction under mild conditions^30^. This system is safer than ultraviolet (UV) light irradiation since UV overexposure induces free radicals and oxidative stress, causing DNA damage and ultimately malignant melanoma^31^. Moreover, chemically modified gelatin, collagen, and hyaluronic acid polymers have been used in photocrosslinked hydrogels because visible light can easily achieve their polymerization. Previous studies using methacrylated gelatin have demonstrated its capacity for wet wound sealing and accelerated polymerization^32,33^. However, methacrylic acid, a constituent material of methacrylated gelatin, is flammable and vaporizes easily, making its preparation hazardous, and it is produced as a degradation product of methacrylated gelatin causing skin and respiratory irritation and mild oral toxicity. In contrast, the natural form of gelatin is safer for applications in the human body. Moreover, natural gelatin is suitable as a hydrogel wound dressing due to its capacity for the sustained, long-term release of various cell growth factors, such as basic fibroblast growth factor (bFGF)^34,35^. bFGF plays a crucial role in the wound healing process by promoting fibroblast proliferation, inducing neovascularization, and increasing collagen synthesis^36^. The topical application of human recombinant bFGF is effective in wound healing in clinical settings^37,38^, however, its frequent application is required for wound healing because of its short half-life in the body.

Therefore, the production of a gelatin hydrogel made of natural gelatin, crosslinked by visible light, will enable its prolonged application to uneven wound surfaces, owing to its flexible shape, along with the administration of cell growth factors, such as bFGF, for tissue regeneration. This study reports a biomimetic tissue-adhesive gelatin hydrogel (hence referred to as hydrogel) capable of forming and adhering within seconds and binding to wet tissue surfaces following visible light irradiation. Our data suggest that this photocrosslinked gelatin hydrogel can promote wound healing and improve skin flap survival.

## Results

### Hydrogel characterization

**Figure 1:**
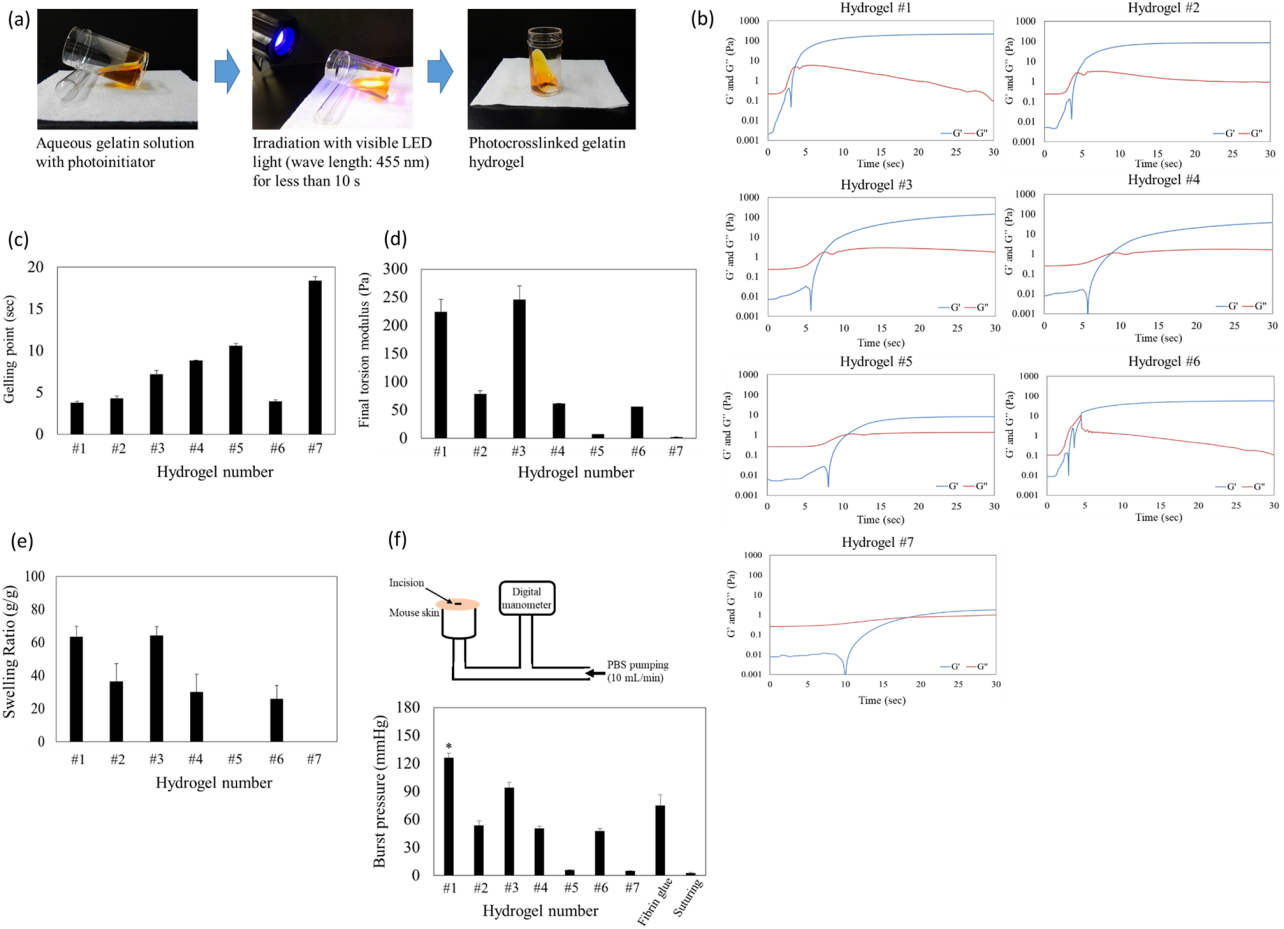
Formation and characterization of hydrogel crosslinked by light irradiation. (**a**) Process of hydrogel formation by photocrosslinking. (**b**–**e**) Mechanical properties of the photocrosslinked gelatin hydrogels. (**b**) To monitor the gelling process, a dynamic time-sweep rheological analysis was conducted using an in situ photo-rheometer (HAAKE Mars and LED 455 nm) showing the formation kinetics for photocrosslinked gelatin hydrogels. (**c**) Gelling points and (**d**) the final torsion moduli, G′, of different hydrogels. The exposure time for all gelling measurements was 120 s (error bars, mean ± SD; *n* = 3 per group). (**e**) Swelling ratios of different hydrogels (*n* = 8 per group) after 24 h incubation in PBS at 37 °C (error bars, mean ± SD). (**f**) Burst pressure of photocrosslinked gelatin hydrogels and fibrin glues. Schematic illustration of the experimental procedure and pressure chamber for burst adhesion testing using punctured and then sealed mouse skin tissue. The observed burst pressure of photocrosslinked gelatin hydrogels and fibrin glue were measured (mean ± SD; *p < 0.005 against fibrin glue; *n* = 5 per group).

The hydrogel and LED power density components are summarized, and the hydrogel code number is shown in Table 1. The gel formed in less than 10 s when exposed to visible light (wavelength, 455 nm; Fig. 1a). The gelling process was monitored using a dynamic time-sweep rheological analyzer with a photo-rheometer (Fig. 1b). Figures 1c shows that the gelling point (the intersection of G′ and G″ curves in Fig. 1b) was at 3.79 ± 0.18 s (hydrogel #1), 4.25 ± 0.31 s (hydrogel #2), and 3.94 ± 0.19 s (hydrogel #6) These gelling points were lower than those for the formation of hydrogels #3, #4, #5, and #7, which took 7.15 ± 0.46 s, 8.84 ± 0.04 s, 10.58 ± 0.32 s, and 18.35 ± 0.55 s, respectively. The final torsion modulus after complete gelation was 224.36 ± 21.65 Pa for hydrogel #1 and 246.10 ± 24.24 Pa for hydrogel #3, which was higher than the shear resistance of 78.97 ± 5.52 Pa for hydrogel #2, 62.03 ± 0.19 Pa for hydrogel #4, 7.19 ± 0.11 Pa for hydrogel #5, 55.92 ± 0.28 Pa for hydrogel #6, and 2.16 ± 0.11 Pa for hydrogel #7 (Fig. 1d). When the photoinitiator concentration was 0.25 mM (hydrogel #5) or gelatin concentration was 5 wt% (hydrogels #6 and #7), the rigid hydrogel was unformed, even when irradiated with 30 mW/cm^2^, and even if gelation occurred, the gel point was found to be very high, with a very small final torsion modulus. From these measurement results, rigid hydrogels #1 or #3 should be more suitable for in vivo applications.

**Table 1:** The hydrogel and LED power density components.

Hydrogel #	Gelatin conc. (wt%)	Photoinitiator conc. (mM)	LED power density (mW/cm^2^)
1	10	1	30
2	10	0.5	30
3	10	1	10
4	10	0.5	10
5	10	0.25	30
6	5	1	30
7	5	0.5	30

Figure 1e shows the maximum SRs, which were achieved after 24 h. Hydrogels #2, #4, and #6 were more brittle than hydrogels #1 and #3 and were difficult to handle. Hydrogels #5 and #7 were unmeasurable because they collapsed during swelling.

Since the adhesion strength of the hydrogel is one of the most important factors for tissue adhesion, its analysis is fundamental^28^. Burst pressure experiments are required to investigate the capacity of these hydrogels to withstand the peeling force from the tissue while rapidly adhering to tissue walls. Figure 1f shows a comparison between the capacity of hydrogels and fibrin glues to adhere to biological surfaces and resist bursting pressures. Hydrogels were formed in situ on the wet surface of mouse subcutaneous tissue, covering a 2-mm diameter hole, placed in a chamber linked to a syringe. Visible light irradiation for 30 s completely polymerized the native gelatin hydrogels, following which the syringe began to pump PBS (pH 7.4, 37 °C) solution and exert pressure on the hydrogel-sealed hole. The measured burst pressure of the photocrosslinked gelatin hydrogel #1 was 126.20 ± 4.74 mmHg, which is higher than that of commercially available surgical fibrin glues (75.00 ± 11.48 mmHg), as shown in Fig. 1f. Hydrogels with a peel force below this pressure cannot be peeled from the tissue. Moreover, the burst pressure of the hydrogel casing seals was higher than normal blood pressure, which would also make it a promising sealing material for bleeding wounds. The burst pressure tests showed that hydrogels were punctured directly above the sealed hole. Although burst pressure punctured the hydrogels, they were not separated from the mouse subcutaneous tissue. This indicated that the tissue adhesion of hydrogels was higher than its mechanical strength and adhesive hydrogels have sufficient internal strength and ability to form strong bonds with tissue substrates. Since hydrogel #1 was the most suitable for applications in the human body and was, therefore, selected for the following experiments.

### Biocompatibility and biodegradation of hydrogel

**Figure 2:**
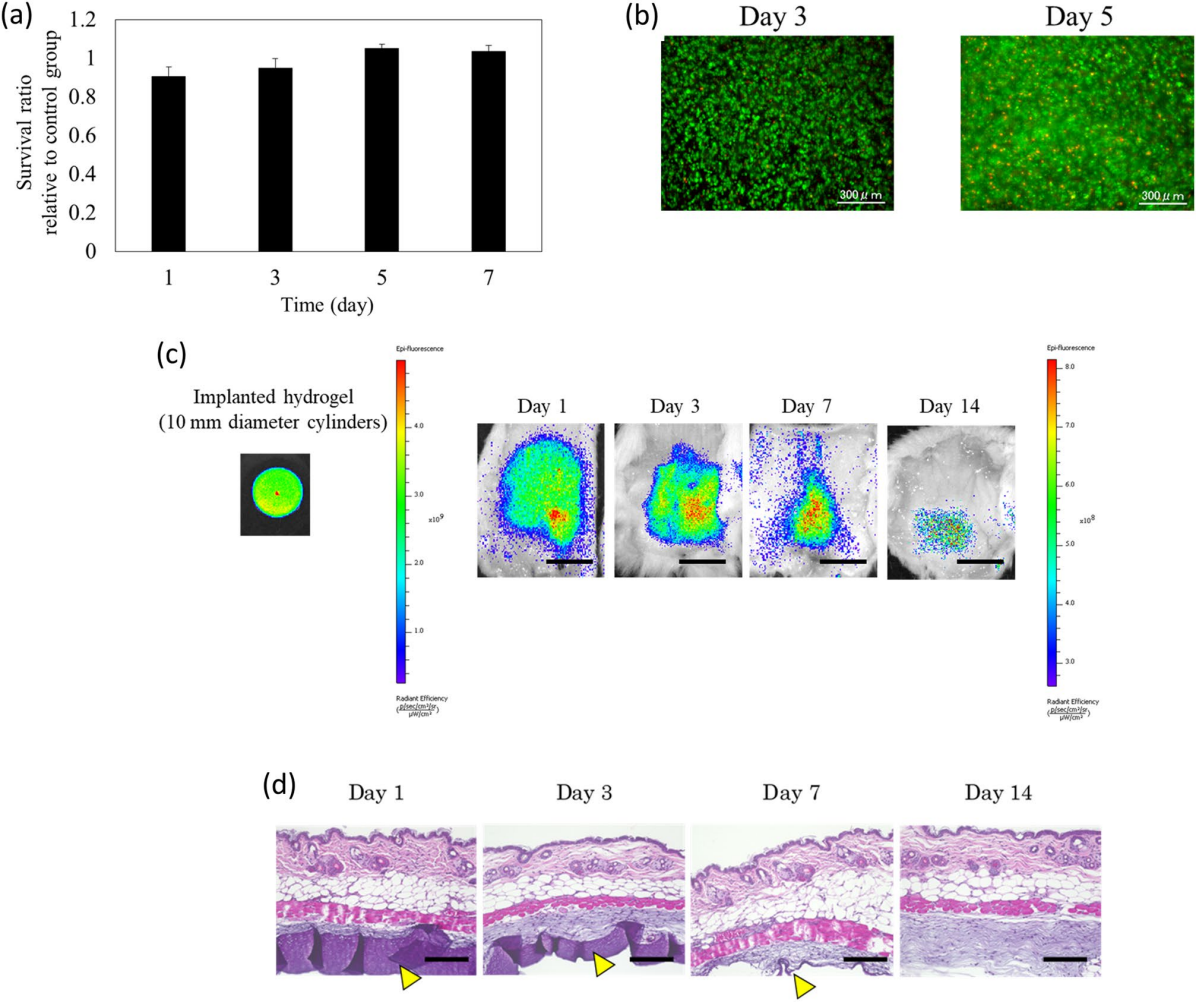
Evaluation of in vitro and in vivo biocompatibility of photocrosslinked gelatin hydrogels. (**a**) Cytotoxicity towards L929 fibroblasts after incubation with hydrogel extracts for 1, 3, 5, and 7 days (*n* = 8 per group). Experimental data were represented by dividing each value by that of the control group (incubated with normal culture medium) for each day (error bars, mean ± SD). (**b**) Live/dead staining of L929 fibroblasts encapsulated in the hydrogels after 3 and 5 days of incubation. L929 fibroblasts were stained with calcein-AM to detect living cells (green) and ethidium homodimer-1 to detect dead cells (red). Scale bar: 300 μm. (**c**) Macroscopic appearance of excised photocrosslinked gelatin hydrogel #1 implants after 1, 3, 7, and 14 days of implantation (*n* = 5 per group). At designated time intervals, the fluorescence of the remaining hydrogel in mouse subcutaneous tissue was imaged using the IVIS Lumina XR, and the fluorescence intensity was analyzed with Living Image Software (PerkinElmer Inc., MA). Scale bar: 10 mm. (**d**) After imaging, the subcutaneous tissues were processed for histological analyses. Pathological staining with hematoxylin and eosin showed several inflammatory cells near the hydrogel–tissue interface on day 1, indicating a post-operative inflammatory response. However, this inflammatory response disappeared within 3 days. The yellow arrowhead indicates the remaining hydrogel. Scale bar: 200 μm.

The cellular cytotoxicity of these hydrogels was evaluated by using L929 mouse fibroblasts. The hydrogel components were did not affect cell growth (Fig. 2a). Moreover, most cells encapsulated in hydrogels survived and stained normally (Fig. 2b).

Next, this hydrogel was subcutaneously implanted into the backs of mice to assess its in vivo biodegradation, the host’s cellular immune response to the implant, and the distribution and persistence of the hydrogel and photoinitiator in each organ. The implanted hydrogel was excised at 1, 3, 7, and 14 days. The hydrogel implant size increased after implantation due to initial osmotic swelling and then decreased with time as the hydrogel was degraded (Fig. 2c). The proportion of implanted hydrogel left after 3 days was 62.11 ± 8.14%, which was reduced to 37.44 ± 3.83% after 7 days and to 8.88 ± 1.98% after 14 days. These results indicate that the hydrogel degraded progressively in vivo. Wound-dressing materials must not be toxic or highly inflammatory^39,40^. Pathological staining with hematoxylin and eosin (Fig. 2d) showed that cell accumulation and tissue damage were not observed around the hydrogel. These results indicate good biocompatibility following hydrogel implantation. There was no fluorescence from the hydrogel or photoinitiator during the evaluation of fluorescence intensity in each organ (liver, kidney, gastrointestinal tract, brain, lung, and spleen) of the mouse according to our preliminary study. This indicates that the degradation products of the hydrogel and photoinitiator were excreted.

### In vivo release profile of bFGF from hydrogel

**Figure 3:**
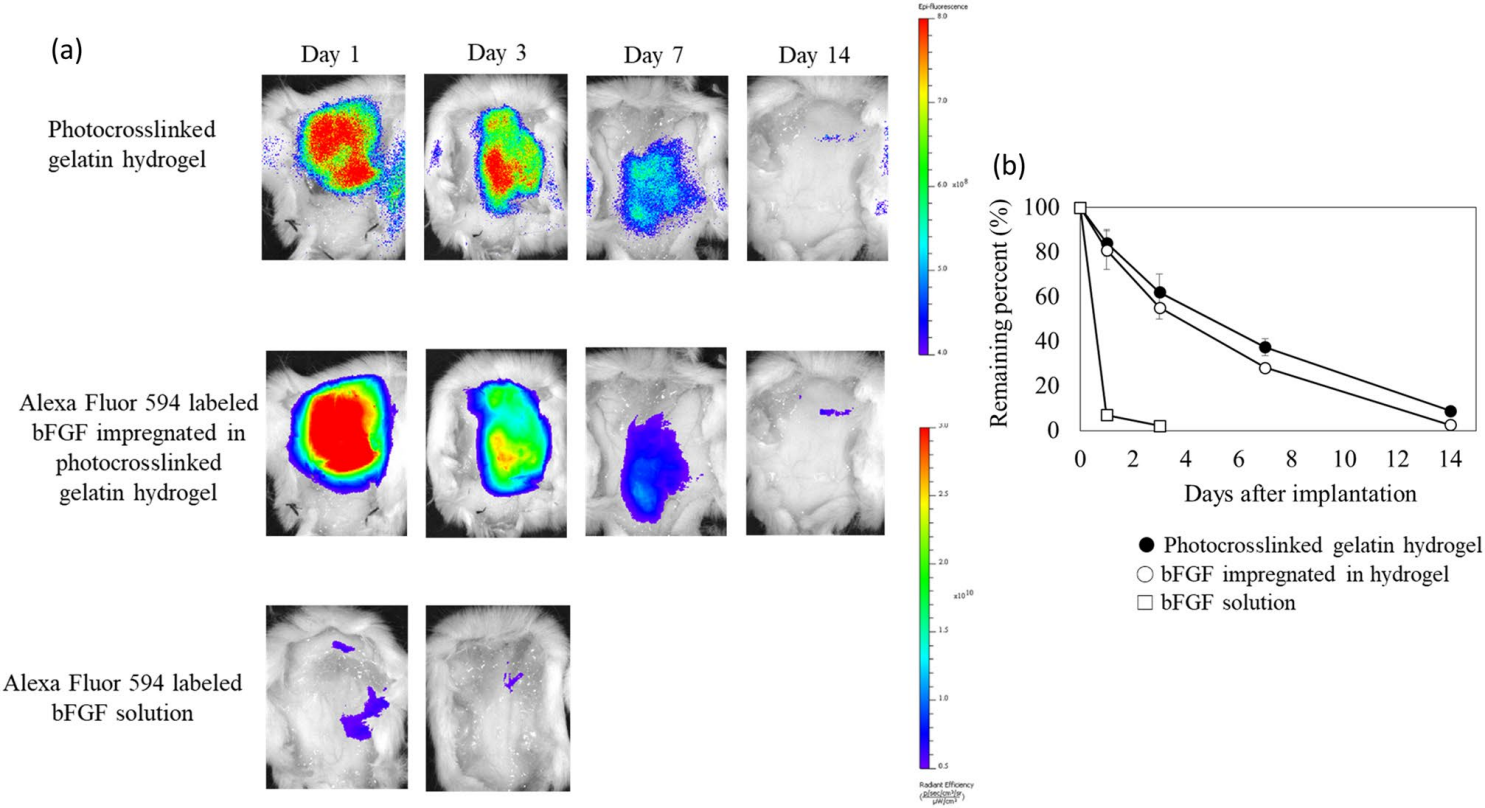
In vivo release of bFGF from photocrosslinked gelatin hydrogel. (**a**) Decreasing patterns of bFGF impregnated in photocrosslinked gelatin hydrogel after subcutaneous implantation into mice. The residual bFGF in the photocrosslinked gelatin hydrogels decreased with implantation time. In contrast, the aqueous solution of bFGF (without gelatin hydrogel) rapidly disappeared from the injected site within 3 days. (**b**) The time profile of bFGF retention was in good accordance with that of hydrogel degradation. These findings indicate the possibility that bFGF is released from the photocrosslinked gelatin hydrogel in the body due to hydrogel biodegradation.

Figure 3a shows the decreasing patterns of bFGF impregnated in the hydrogel after subcutaneous implantation in mice. The residual bFGF in the hydrogels appeared to decrease with implantation time. In contrast, the aqueous solution of bFGF (without gelatin) disappeared rapidly from the injected site within 3 days. These results, depicted in Fig. 3b, indicate that bFGF retention was temporally linked to hydrogel degradation, suggesting the possibility that bFGF is released from the hydrogel in the body due to hydrogel biodegradation^35^.

### Wound healing in diabetic mice by sustained release of bFGF

**Figure 4:**
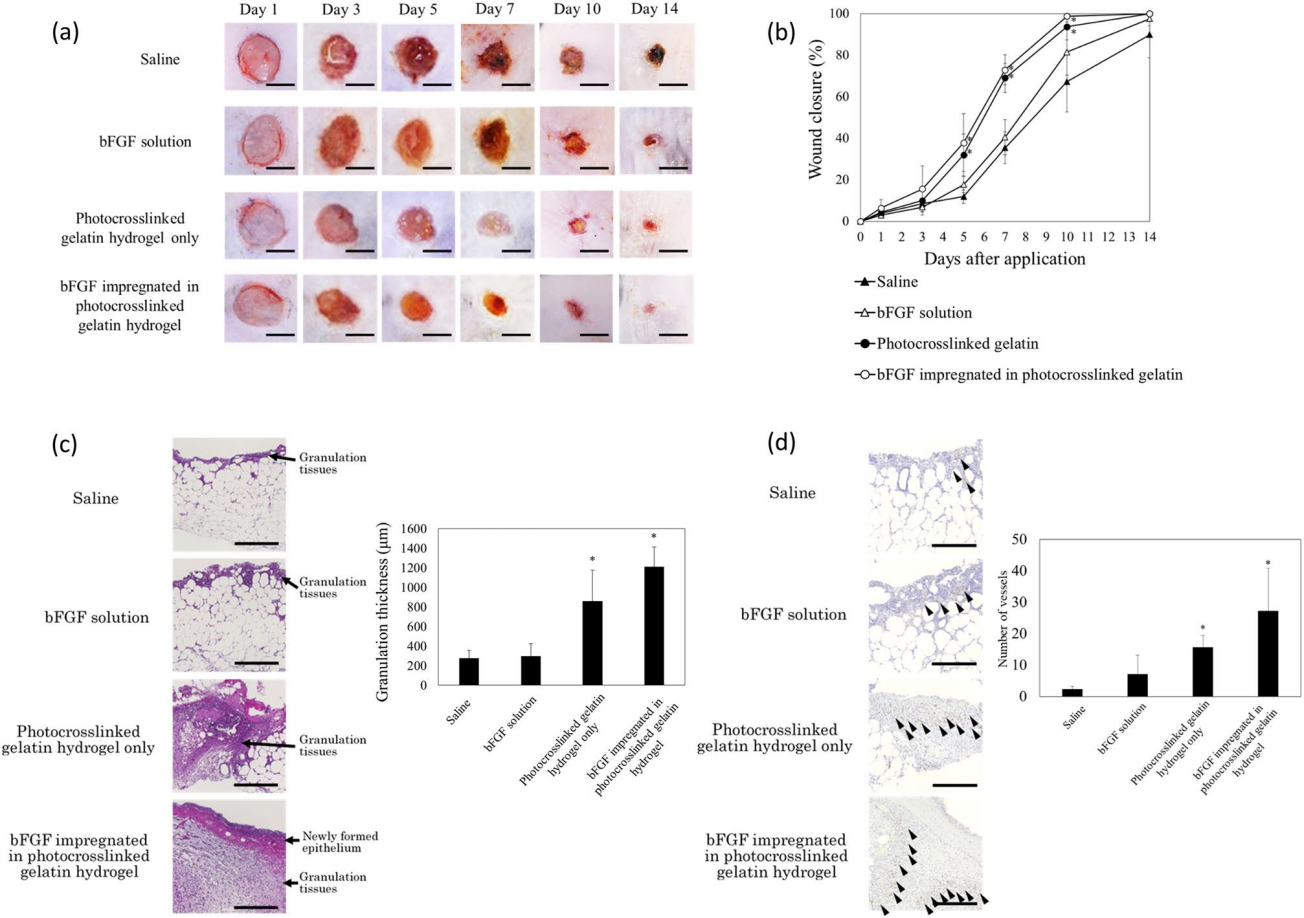
Time course of wound closure and histological evaluation in diabetic mice after sustained release of bFGF from photocrosslinked gelatin hydrogel. (**a**) Images of in vivo wound closure studies for saline, bFGF solution (Fiblast spray), and photocrosslinked gelatin hydrogels with and without bFGF. Macroscopic photographs of wounds at days 5, 7, and 10 demonstrated significantly faster wound closure in the group treated with photocrosslinked gelatin hydrogels with bFGF. Scale bar: 5 mm. (**b**) Average wound closure from days 0 to 14 [expressed as a percent of the day 0 control (100%)]. On day 7, the group treated photocrosslinked gelatin hydrogels with bFGF showed a greater extent of wound closure when compared to that in saline and bFGF solution groups. *p < 0.005 vs saline group. (**c**) Histology of wounds treated with saline, bFGF solution (Fiblast spray), and photocrosslinked gelatin hydrogels with and without bFGF on post-operative day 7. The surfaces of the wounds treated with the photocrosslinked gelatin hydrogels with bFGF showed epithelium neoformation. This wound also showed the formation of granulation tissue with the highest thickness. The increased number of fibroblasts and the collagen amount in the wounds treated with bFGF-containing photocrosslinked gelatin hydrogels significantly contributed to enhanced granulation tissue formation. Scale bar: 300 μm; *p < 0.005. (**d**) Histological evaluation of the blood capillaries. The wounds treated with photocrosslinked gelatin hydrogels with bFGF had an increased distribution of blood capillaries (arrowhead) compared to that with saline and bFGF solution (Fiblast spray) on post-operative day 7. Scale bar: 300 μm; *p < 0.005 vs saline group.

The wound closure efficacy of hydrogels with bFGF was determined in vivo by creating full-thickness skin incisions on the backs of diabetic mice (db mice, C57BLKS/J Iar-+ Lepr^db^/ + Lepr^db^), followed by the application of hydrogels at the wound sites. Figure 4a shows the images of the wound taken at different time intervals after treatment with control (saline), bFGF aqueous solution (Fiblast spray), and hydrogels with and without bFGF. There was no sign of inflammation or infection in the hydrogel-covered wounds at any time point. Further, the growth of the new epidermis extended to the wound center with all treatment conditions, thereby reducing the wound area. Among treatment groups, hydrogels with bFGF showed the most accelerated wound contraction up to 10 days compared to that with saline, bFGF solution, and hydrogels without bFGF, indicating that treatment with bFGF-containing hydrogels improved and accelerated wound healing. Moreover, the wounds of all mice in the saline group and some mice in the bFGF solution group formed scabs, whereas those of mice in the hydrogel groups did not. Seven days post-transplantation, the wound closure percentage of the wounds treated using hydrogels with bFGF was approximately 72.9%, whereas that of the wounds treated with saline, bFGF solution, and hydrogels without bFGF showed lower wound closure rates (35.4%, 40.5%, and 69.0%, respectively; Fig. 4b).

Figure 4c shows the histology of wounds covered with hydrogels with bFGF, as well as other treatments, on post-operative day 7. The surface of the wounds treated with the hydrogels with bFGF showed the formation of new epithelium, as observed with H&E staining (Fig. 4c). The assessment of the density of fibroblasts revealed that the hydrogels with bFGF induced the highest fibroblast density in the treated wounds. In addition, in these wounds, granulation tissue with the highest thickness was observed (Fig. 4c). The increased number of fibroblasts and the amount of collagen in the wounds treated with bFGF-containing hydrogels significantly contributed to enhanced granulation tissue formation. Further, wounds covered with hydrogels with bFGF showed densely packed, parallel collagen fibers in the extracellular matrix. In contrast, loosely packed, irregular collagen fibers were observed in the other treatment groups. The histological evaluation of the blood capillaries revealed that the wounds treated with bFGF-containing hydrogels had an increased distribution of blood capillaries compared to that of other treatment groups (Fig. 4d).

### Effect of hydrogels on skin flap survival

**Figure 5:**
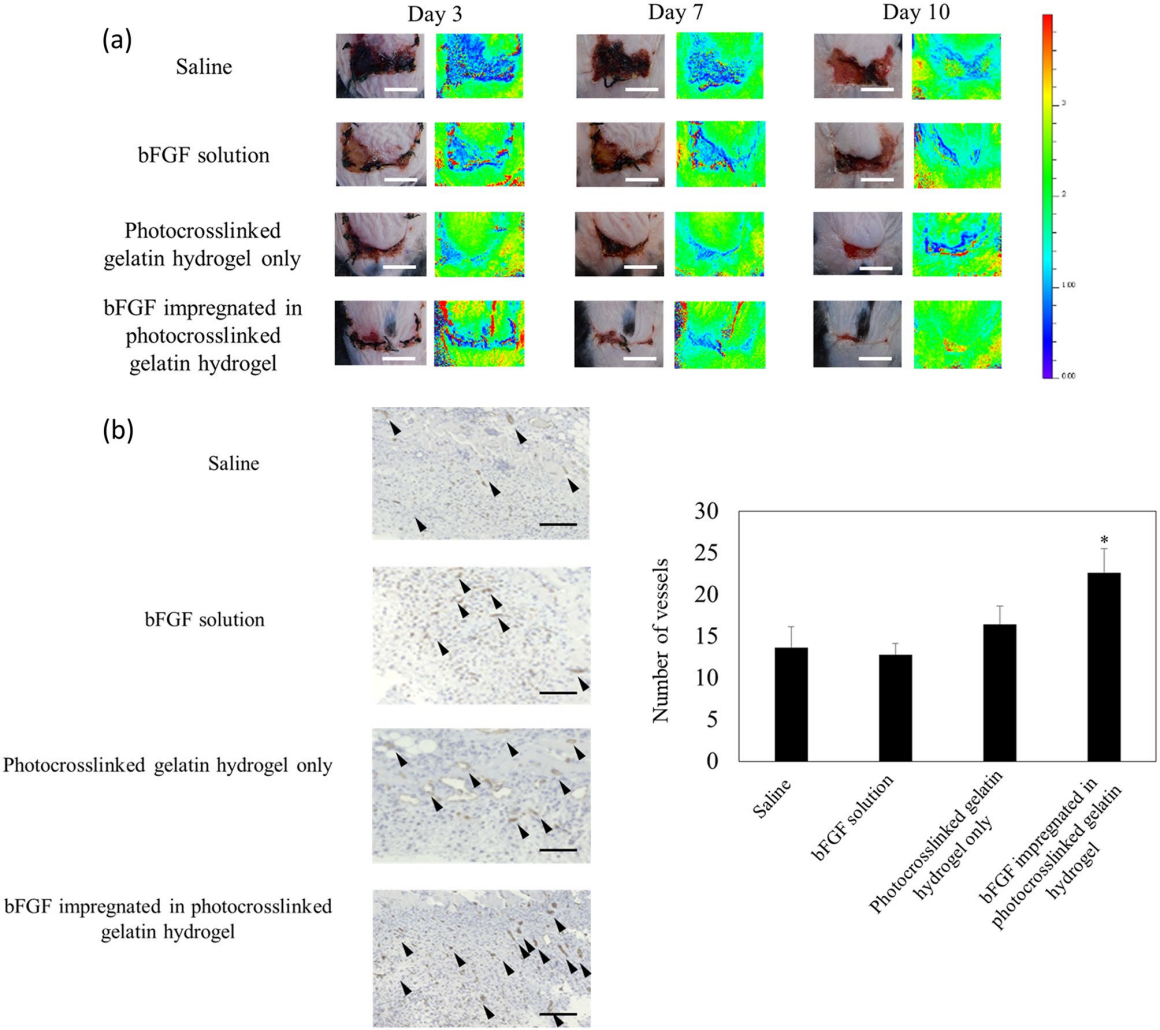
Improved flap survival in mice by sustained release of bFGF from photocrosslinked gelatin hydrogel. (**a**) Comparison of survival of random pattern skin flaps among saline, bFGF solution (Fiblast spray), and photocrosslinked gelatin hydrogels with and without bFGF on post-operative day 10. Assessment of tissue viability at the random skin flap area in both groups of transgenic mice expressing FRET-based biosensors, named “ATeam,” for visualization of ATP levels in living cells. Representative images showing the ATP level in the skin flap was greater in the photocrosslinked gelatin hydrogel with bFGF group on post-operative day 10 than in the other groups. Scale bar: 10 mm. The color bar shows the FRET ratio. The higher the FRET ratio, the higher the intracellular ATP content, as detected using ATeam. (**b**) Histological evaluation of the blood capillaries. Flaps treated with bFGF-containing photocrosslinked gelatin hydrogels had an increased distribution of blood capillaries compared to that with saline and bFGF solution (Fiblast spray) on post-operative day 10. Scale bar: 100 μm; *p < 0.005 vs saline group.

The survival of the random-pattern skin flap tissue of mice was evaluated on day 10 post-operation (Fig. 5a). Living transgenic mice expressing FRET-based biosensors, “ATeam,” were used to study the dynamics of ATP in vivo, conducting assays to elucidate the physiological maintenance of energy. By confirming ATP levels, tissue survival can be assessed at the cellular level^41,42^. While applying the skin flap on the backs of the mice, hydrogels were molded subcutaneously via light irradiation according to the shape of the skin flap. Tissue necrosis due to ischemia occurs from the end of the skin flap. The survived end of the skin flap area was significantly larger in the bFGF-containing hydrogel group as compared to that in other groups on day 10, which suggests that cell survival is maintained at the tip of the flap as well, which contributes to improving the survival rate and viability of the skin flap. Histological evaluation revealed that the numbers of blood capillaries in the skin flaps of the hydrogel with bFGF group indicated an increased numbers of blood capillaries compared to that in other groups (Fig. 5b).

## Discussion

This study, to the best of our knowledge, is the first to demonstrate that diabetic wound healing and survival rates of skin flaps can be enhanced using hydrogels with rapid gelling and sustained bFGF release properties. bFGF-containing hydrogels have a great potential use for wound healing and skin flap formation. The increase of fibroblast proliferation indicates that hydrogels combined with bFGF may promote cell migration, improve wound healing, and skin flap formation. Wound closure experiment indicates that hydrogels containing bFGF may significantly enhance wound epithelialization, collagen deposition, and accelerate wound healing. The hydrogel without bFGF showed significantly better wound closure than the saline group. Thus, both hydrogel and bFGF accelerated wound healing in vivo. This could be attributed to the material properties of the hydrogel. These properties may maintain a physiologically moist microenvironment and adherence to the wound site, thereby enhancing the wound closure that occurred earlier than the control. Moreover, bFGF promotes fibroblast proliferation, which could also contribute to the generation of the contractile force at the wound area during the initial stages of wound healing^43^. Furthermore, the wounds of all mice in the saline group and some mice in the bFGF solution group formed scabs, whereas those of mice in the hydrogel groups did not. As scab formation has been reported to delay the promotion of epithelialization, wound treatment with hydrogels that do not allow scab formation can promote wound healing^44^. Therefore, hydrogels with bFGF hold great promise in wound healing and skin flap applications.

Although some studies have reported other materials that can adhere to wet tissue surfaces^28,29,45,46^, it is difficult to completely cover uneven and oddly shaped wound surfaces with pre-molded biomaterials, compromising wound healing and reducing the survival rate of the skin flap. Our flexible hydrogel produced at the wound site by visible light using natural gelatin can be applied to uneven wound surfaces, where it promotes healing through the long-term release of cell growth factors. Photocrosslinked gelatin is especially promising owing to rapid photoactivation. In the photocrosslinking reaction of gelatin molecular chains, the hydroxyl groups of amino acid side chains form free radicals (hydroxyl radical), and the reaction proceeds^47^. Since there are reactive groups in gelatin molecules, the gelation reaction is rapid (Fig. 1c). Since the amount of generated free radicals differs depending on the concentration of the photoinitiator and the LED power density, there might have been a slight difference in gelation time between the hydrogel. However, at present, the most commonly used photocrosslinking initiator, Irgacure 2959 (I2959), is oxygen-sensitive and requires the airtight protective barriers. Moreover, to activate I2959, UV light is potentially damaging to cellular DNA, and the gelation reaction with I2959 is slower than that with the ruthenium compound used as a photoinitiator in this study^48^. UV irradiation of cells generates reactive oxygen species (ROS), which causes cytotoxicity. The amount of ROS generated by cell irradiation with visible light used in this study (wavelength: 455 nm, 30 mW/cm^2^ for 30 s) was below the detection limit (data not shown). Moreover, the photocrosslinking using ruthenium compounds offers a significantly deeper light penetration depth compared to the I2959, allowing thick hydrogels to be fabricated with homogenous crosslinking density throughout the construct^49^. Past reports using methacrylated gelatin outlined its capacity for wet wound adherence and accelerated polymerization^32,33,50,51^. However, methacrylic acid, a constituent material of methacrylated gelatin, is flammable and vaporizes easily, hence require special attention during preparation. Moreover, it is a degradation product of methacrylated gelatin, which causes skin and respiratory irritation and a small amount of oral toxicity. Therefore, natural gelatin is safer for clinical applications than chemically modified gelatin. In this study, photocrosslinked gelatin hydrogels were imparted with properties such as fast wound adherence, strong adhesion to wet tissue surface, greater biocompatibility, and sustained, long-term bFGF release. However, the processes involved in developing such hydrogels are not simple.

The three physiological stages of wound healing are inflammation, tissue regeneration, and tissue remodeling^52^. Growth factors, such as bFGF, vascular endothelial growth factor (VEGF), epidermal growth factor (EGF), and platelet-derived growth factor (PDGF), play important roles at various stages^53^. In this study, bFGF, a primary promoter of cell proliferation during wound healing^54^, was selected to enhance wound healing and survival of the skin flap in hydrogel-treated wounds. However, bFGF loses its bioactivity under normal physiological conditions. Therefore, it is essential to incorporate bFGF into a sustainable drug release system, such as hydrogel and control its release to increase the efficiency of bFGF utilization^35^.

Flaps are one of the most essential tools used by plastic surgeons. Blood perfusion is the major factor limiting the size of random-pattern flaps. Vascularization is critical for the survival of the flap. Hence, developing tools to enhance angiogenesis and blood flow in the flap tissue remains essential.

Although this study provides some credible results, it has two main limitations. First, mouse skin differs from that of humans in the presence of the panniculus carnosus muscle layer. Therefore, a direct comparison of random-pattern skin flaps between mice and humans is difficult. Further research is therefore warranted to determine whether hydrogel treatment can increase the survival area in larger flaps. Second, the cytotoxicity of the photoinitiator, pentamethylcyclopentadienyl triphenylphosphine ruthenium chloride, needs evaluation. Our results revealed no apparent cytotoxicity at the cellular level. A thorough examination of the H&E-stained organ sections revealed no significant signs of toxicity. Moreover, the degradation products of the hydrogel and photoinitiator were excreted, without accumulating in the body. Although further safety tests are required to apply this hydrogel to clinical-phase testing, these results further support the potential of hydrogels with bFGF for use in diabetic wound healing and skin flap applications.

The precursor of this hydrogel has a relatively low viscosity and can be injected. Moreover, a flashlight with a visible light spectrum or an optical fiber can enable the potential use of this material for completely suture-less adherence of skin flaps, making it a promising surgical bio-glue. Based on our results, bFGF-containing photocrosslinked gelatin hydrogels are promising biomaterials for clinical use in wound healing owing to their ease of usage, high efficacy, and sustainability.

## Materials and methods

### Photocrosslinked gelatin hydrogel preparation

Gelatin (beMatrix, Nitta Gelatin, Inc., Japan) was dissolved in Milli-Q ultrapure water at 10 or 5 wt% at 37 °C. The visible light photoinitiator, pentamethylcyclopentadienyl triphenylphosphine ruthenium chloride, and sodium persulfate (10 mM; Advanced BioMatrix, Inc., CA) were gently mixed with gelatin solution immediately before use. LED (Thorlab, Inc., wavelength: 455 nm) was used for the light source.

### Rheological properties

Rheological properties of hydrogels were analyzed as previously described^55^. Briefly, dynamic rheology experiments were performed using a HAAKE MARS photo-rheometer (Thermo Fisher Scientific, Inc.) with parallel-plate geometry and an LED light source at 37 °C. Time-sweep oscillatory tests of the mixture of gelatin with the photoinitiator were performed (*n* = 3). Strain sweeps were performed on the pre-gel solution to verify the linear response. The gelling point was determined at the time when the torsion modulus (G′) surpassed the loss modulus (G″).

### Swelling ratio of hydrogels

The hydrogels (*n* = 8) were incubated in PBS at 37 °C for 24 h and then lightly blotted dry and weighed (Ws). Hydrogels were then freeze-dried and weighed to determine the dry weight (Wd). The swelling ratio (SR) of the swollen gel was calculated as follows^56^:

$$\mathrm{SR}=\frac{(\mathrm{Ws}-\mathrm{Wd})}{\mathrm{Wd}}.$$

### Burst pressure test

The burst pressure test was performed as previously described^57^. Briefly, a piece of 2 × 2 cm^2^ ICR mouse skin was cut. The skin was fixed to the measurement device linked to a syringe filled with PBS (pH 7.4, 37 °C). A 2-mm incision was made on the skin surface, and the surface was kept wet. Then, 500 μL of the gelatin and photoinitiator mixture was applied onto the incision of the wet surface of mouse subcutaneous tissue, after which the hydrogels formed in situ at the puncture site after visible light (wavelength: 455 nm, 30 mW/cm^2^) illumination. The thickness of the hydrogels was ~ 3 mm, and burst pressure was measured after gel formation. Peak pressure before pressure loss was considered the burst pressure. All measurements were repeated five times to ensure replicability. Fibrin Glue (Bolheal, Teijin Pharma, Limited, Japan) was tested using the same parameters and conditions.

### Cytotoxicity test of hydrogel

The proliferation of L929 mouse fibroblast cells was assessed using a DNA assay. Briefly, L929 cells were seeded into 96-well plates at a density of 1000 cells/100 μL/well and cultured in Dulbecco’s modified Eagle’s medium (DMEM; Thermo Fisher Scientific, Waltham, MA, USA) containing 10% fetal bovine serum (FBS; Thermo Fisher Scientific) for 24 h at 37 °C in a 5% CO_2_ humidified incubator to obtain a monolayer of cells. Gelatin, pentamethylcyclopentadienyl triphenylphosphine ruthenium chloride, and sodium persulfate was dissolved in DMEM with FBS at final concentration of 10 wt%, 1 mM, and 10 mM respectively. The culture medium was replaced with hydrogel components in culture medium (100 μL/well), and cells were further incubated for 1, 3, 5 and 7 days. The sample solution was removed, and cells were carefully washed using PBS (pH 7.4, 37 °C) three times, and their numbers were determined using a Hoechst 33342 based DNA assay kit (Dojindo, Japan) according to the instruction manual. Fluorescence was measured using a microplate reader (SpectraMax iD5, Molecular Devices, CA) at Ex: 350 nm and Em: 461 nm. For each sample, eight independent cultures were prepared, and proliferation assays were repeated three times for each culture. Experimental data were represented by dividing each value by that of the control group (incubated with normal culture medium) for each day.

In addition, gelatin and a photoinitiator mixture containing L929 cells (100 μL) was irradiated (30 mW/cm^2^, 30 s) to produce cell-laden hydrogels, and cells were cultured in DMEM with 10% FBS at 37 °C with 5% CO_2_ for 3 and 5 days. Cell viability was determined using the live/dead cytotoxicity staining kit (Dojindo, Japan). By incubating the cells with this reagent for 60 min, the cells in the hydrogel were stained. Encapsulated cells were imaged under a fluorescence microscope (BZ-9000, Keyence, Japan).

### In vivo biocompatibility of hydrogel

All animal experiments performed for this study were carried out in compliance with the ARRIVE guidelines and the protocol was approved by the Committee on the Ethics of Animal Experiments of National Defense Medical College (approved numbers: 19010 and 19065). All methods were performed in accordance with relevant guidelines and regulations of National Defense Medical College. Female ICR mice (~ 30 g) were used for in vivo biocompatibility studies. A 1 cm incision was made in the mediodorsal skin of the mice, and a lateral subcutaneous pocket was prepared. Hydrogel samples (10 × 1 mm cylinders) were implanted under sterile conditions. At designated time intervals (days 1, 3, 7, and 14), mice (*n* = 5 for each day) were sacrificed, and the fluorescence of the remaining hydrogel in mice subcutaneous tissue was imaged using the IVIS Lumina XR (Ex: 460 nm, Em: 620 nm), and the intensity of fluorescence was analyzed using Living Image Software (PerkinElmer Inc., MA) and were processed for histological analyses.

### In vivo hydrogel degradation and sustained release of bFGF

bFGF (Fujifilm Wako pure Chemical Corp., Japan) was desalted and fluorescently labeled using the Alexa Fluor 594 Microscale Protein Labeling Kit (Thermo Fisher Scientific, Inc.) according to the manufacturer’s instructions. The absorption spectrum of Alexa Fluor 594 is completely different from that of the hydrogel, meaning that they do not interfere with each other during fluorescence imaging. Female ICR mice (~ 30 g) were used to study in vivo degradation and bFGF sustained release from a hydrogel. A 1 cm incision was made in the mediodorsal skin of mice, and a lateral subcutaneous pocket was prepared. Hydrogel with 5 μg bFGF samples (10 × 1 mm cylinders) was implanted under sterile conditions. Alexa Fluor 594-labeled bFGF aqueous solution was used as a control group (10 μg in PBS, pH 7.4) and was subcutaneously injected. At designated time intervals (days 1, 3, 7, and 14), mice (*n* = 5 for each day) were sacrificed. Fluorescence of the remaining hydrogel and bFGF on mouse subcutaneous tissue were imaged using the IVIS Lumina XR (excitation, 460 nm and emission, 620 nm for hydrogel imaging; excitation, 580 nm and emission, 620 nm for Alexa Fluor 594-labeled bFGF imaging), and the intensity of fluorescence was analyzed using Living Image Software (PerkinElmer Inc., MA).

### Wound healing in diabetic mice through the sustained release of bFGF

The wound closure efficacy of the photocrosslinked gelatin hydrogels with bFGF was determined in vivo by creating full-thickness skin incisions on the backs of diabetic mice (db mice, C57BLKS/J Iar-+ Lepr^db^/ + Lepr^db^, 8 weeks of age, purchased from Nihon SLC). Under anesthesia, these mice were shaved and depilated. Cutaneous wounds were created as previously described^58^. Immediately after creating the wound, the following treatments were applied topically on the wound for different experiment groups (*n* = 5 per group): Group A, saline solution; Group B, conventional method of Fiblast spray (bFGF solution; 1 µg/cm^2^/day) without gelatin; Group C, gelatin and photoinitiator without bFGF were photocrosslinked on the wound; Group D, gelatin and photoinitiator with bFGF (7 µg/cm^2^) were photocrosslinked on the wound. Groups C and D were photocrosslinked through illumination with visible light (wavelength: 455 nm, 30 mW/cm^2^ for 30 s). Film dressing was covered to prevent drying and contamination. At designated time intervals (days 3, 5, 7, 10, and 14), mice (*n* = 5 for each day) were sacrificed. After peeling off the hydrogel from the wound, images of the wound were obtained, and the wound area was evaluated using ImageJ software. The area of the skin defect and the inner diameter were measured at each time point. The skin defect area on day 0 was used as the control (100%) to normalize the ratios of areas obtained from later observations on days 3, 5, 7, 10, and 14^59^. Based on the results of this experiment, the wounds on post-operative day 7, which had the largest difference between Group B and D, were histologically evaluated. The harvested wounds were fixed with a 5% formalin neutral buffer solution and embedded in paraffin. Further, 5 μm-thick sections were cut from the central region of the wound. H&E staining was performed following the standard protocol. Sections were imaged with a BZ-9000 microscope (Keyence, Japan). Paraffin sections were stained for CD31 for microvessel staining followed by standard immunohistochemical staining with DAB to examine the vascular density of the wound post-treatment. Five fields were randomly selected, and CD31-DAB-positively stained vessels were counted using the BZ-2 Analyzer (Keyence, Japan).

### Improved flap survival in mice by sustained release of bFGF

Improvements in skin flap survival mediated by hydrogels with bFGF were determined in vivo by creating a random pattern skin flap on the back of ATP imaging mice. The ATP levels of cells in flap tissues were estimated by expressing FRET-based biosensors in transgenic mice, namely “ATeam,” to visualize ATP levels (ATeam mice were kindly gifted by Dr. Masamichi Yamamoto, Kyoto University, Japan. Papers are in preparation). A modified McFarlane flap^60^ template with a size of 4 × 2 cm^2^ (exceeding a 2:1 length-to-width ratio) was traced using a surgical marker on the dorsal surface of the ATeam mice after shaving and depilating under anesthesia. The mice were then divided into four equal groups (*n* = 5 per group) as follows: Group A, a saline solution was injected subcutaneously; Group B, conventional method of subcutaneous bFGF spray (1 µg/cm^2^/day) without gelatin; Group C, gelatin and photoinitiator without bFGF were molded subcutaneously on demand via light irradiation (wavelength: 455 nm, 30 mW/cm^2^ for 30 s) according to the shape of the skin flap area; Group D, gelatin and a photoinitiator with bFGF (7 µg/cm^2^) were molded subcutaneously on demand via light irradiation (wavelength: 455 nm, 30 mW/cm^2^ for 30 s) according to the shape of the skin flap area. The flaps were visually assessed 10 days after treatment after analyzing digital images of the flaps recorded using a digital camera. The ATP level in the skin flap was imaged by the IVIS Lumina XR, and the FRET ratio was analyzed with Living Image Software (PerkinElmer Inc.).

The flaps were harvested from euthanized animals on post-operative day 10. The harvested wounds were fixed with a 5% formalin neutral buffer solution and embedded in paraffin. Paraffin sections were stained for CD31 for microvessel staining followed by standard immunohistochemical staining with DAB to examine the vascular density of the wound post-treatment. Five fields were randomly selected, and CD31-DAB-positively stained vessels were counted using the BZ-2 Analyzer (Keyence, Japan).

### Statistical analysis

All data are presented as the mean ± SD. Differences between the values were evaluated using one-way analysis of variance (ANOVA, Tukey’s post-hoc test), except in the in vivo biocompatibility experiment where two-way ANOVA with Tukey’s post-hoc test was used. A value of *p* < 0.05 was considered statistically significant.

## Data Availability

The datasets generated during and/or analyzed during the current study are available from the corresponding author on reasonable request.

## References

[1] Guo S, Dipietro LA (2010). Factors affecting wound healing. J. Dent. Res..

[2] Maalej H (2014). Rhelogical, dermal wound healing and in vitro antioxidant properties of exopolysaccharide hydrogel from *Pseudomonas stutzeri* AS22. Colloids Surf. B Biointerfaces.

[3] Lopez AD, Mathers CD, Ezzati M, Jamison DT, Murray CJ (2006). Global and regional burden of disease and risk factors, 2001: Systematic analysis of population health data. Lancet.

[4] Wu J, Zhao X, Wu D, Chu CC (2014). Development of a biocompatible and biodegradable hybrid hydrogel platform for sustained release of ionic drugs. J. Mater. Chem. B.

[5] Yadav S, Sharma AK, Kumar P (2020). Nanoscale self-assembly for therapeutic delivery. Front. Bioeng. Biotechnol..

[6] Fukunaga Y (2017). Topical application of nitrosonifedipine, a novel radical scavenger, ameliorates ischemic skin flap necrosis in a mouse model. Wound Repair Regen..

[7] Lu WW, Ip WY, Jing WM, Holmes AD, Chow SP (2000). Biomechanical properties of thin skin flap after basic fibroblast growth factor (bFGF) administration. Br. J. Plast. Surg..

[8] Rinsch C (2001). Delivery of FGF-2 but not VEGF by encapsulated genetically engineered myoblasts improves survival and vascularization in a model of acute skin flap ischemia. Gene Ther..

[9] Hihara M (2020). Improved viability of murine skin flaps using a gelatin hydrogel sheet impregnated with bFGF. J. Artif. Organs.

[10] Nazarnezhad S, Baino F, Kim HW, Webster TJ, Kargozar S (2020). Electrospun nanofibers for improved angiogenesis: Promises for tissue engineering applications. Nanomaterials (Basel)..

[11] Smandri A (2020). Natural 3D-printed bioinks for skin regeneration and wound healing: A systematic review. Polymers (Basel)..

[12] Broussard KC, Powers JG (2013). Wound dressings: Selecting the most appropriate type. Am. J. Clin. Dermatol..

[13] Francesko A, Petkova P, Tzanov T (2018). Hydrogel dressings for advanced wound management. Curr. Med. Chem..

[14] Gainza G, Villullas S, Pedraz JL, Hernandez RM, Igartua M (2015). Advances in drug delivery systems (DDSs) to release growth factors for wound healing and skin regeneration. Nanomedicine.

[15] Gurtner GC, Werner S, Barrandon Y, Longaker MT (2008). Wound repair and regeneration. Nature.

[16] Miller R, Wormald JCR, Wade RG, Collins DP (2019). Systematic review of fibrin glue in burn wound reconstruction. Br. J. Surg..

[17] Gaspar-Pintiliescu A, Stanciuc AM, Craciunescu O (2019). Natural composite dressings based on collagen, gelatin, and plant bioactive compounds for wound healing: A review. Int. J. Biol. Macromol..

[18] Tavakoli S, Klar AS (2020). Advanced hydrogels as wound dressings. Biomolecules.

[19] Chen X, Jiang Z, Chen Z, Wang D (2011). Application of skin traction for surgical treatment of grade IV pressure sore: A clinical report of 160 cases. Spinal Cord.

[20] Elisseeff J (1999). Transdermal photopolymerization for minimally invasive implantation. Proc. Natl. Acad. Sci. U. S. A..

[21] Dimatteo R, Darling NJ, Segura T (2018). In situ forming injectable hydrogels for drug delivery and wound repair. Adv. Drug Deliv. Rev..

[22] Hou S, Wang X, Park S, Jin X, Ma PX (2015). Rapid self-integrating, injectable hydrogel for tissue complex regeneration. Adv. Healthc. Mater..

[23] Pertici V (2019). Degradable and injectable hydrogel for drug delivery in soft tissues. Biomacromol.

[24] Ren B (2018). Injectable polysaccharide hydrogel embedded with hydroxyapatite and calcium carbonate for drug delivery and bone tissue engineering. Int. J. Biol. Macromol..

[25] Le TMD (2019). Physically crosslinked injectable hydrogels for long-term delivery of oncolytic adenoviruses for cancer treatment. Biomater. Sci..

[26] Lee JH (2018). Injectable hydrogels delivering therapeutic agents for disease treatment and tissue engineering. Biomater. Res..

[27] Alonso JM, Andrade Del Olmo J, Perez Gonzalez R, Saez-Martinez V (2021). Injectable hydrogels: From laboratory to industrialization. Polymers (Basel)..

[28] Li J (2017). Tough adhesives for diverse wet surfaces. Science.

[29] Annabi N (2017). Engineering a highly elastic human protein-based sealant for surgical applications. Sci. Transl. Med..

[30] Ifkovits JL, Burdick JA (2007). Review: Photopolymerizable and degradable biomaterials for tissue engineering applications. Tissue Eng..

[31] Yang DH, Chun HJ (2020). Visible light-curable hydrogel systems for tissue engineering and drug delivery. Adv. Exp. Med. Biol..

[32] Bao Z, Gao M, Sun Y, Nian R, Xian M (2020). The recent progress of tissue adhesives in design strategies, adhesive mechanism, and applications. Mater. Sci. Eng. C Mater. Biol. Appl..

[33] Hong Y (2019). A strongly adhesive hemostatic hydrogel for the repair of arterial and heart bleeds. Nat. Commun..

[34] Tabata Y, Ikada Y (1999). Vascularization effect of basic fibroblast growth factor released from gelatin hydrogels with different biodegradabilities. Biomaterials.

[35] Tabata Y, Nagano A, Ikada Y (1999). Biodegradation of hydrogel carrier incorporating fibroblast growth factor. Tissue Eng..

[36] Gospodarowicz D (1991). Biological activities of fibroblast growth factors. Ann. N. Y. Acad. Sci..

[37] Akita S, Akino K, Imaizumi T, Hirano A (2005). A basic fibroblast growth factor improved the quality of skin grafting in burn patients. Burns.

[38] Kinoshita N (2012). The usefulness of basic fibroblast growth factor for radiation-exposed tissue. Wound Repair Regen..

[39] Annabi N (2014). 25th anniversary article: Rational design and applications of hydrogels in regenerative medicine. Adv. Mater..

[40] Gaharwar AK (2014). Shear-thinning nanocomposite hydrogels for the treatment of hemorrhage. ACS Nano.

[41] Angel MF, Schieren G, Jorysz M, Knight KR, O'Brien BM (1989). The beneficial effect of chlorpromazine on dorsal skin flap survival. Ann. Plast. Surg..

[42] Jurell G, Fredholm BB (1981). Early changes in ATP and cyclic AMP levels in experimental critical skin flaps. Acta Physiol. Scand..

[43] Kaminishi-Tanikawa A (2011). Features of wound healing shown by fibroblasts obtained from the superficial and deep dermis. J. Plast. Surg. Hand Surg..

[44] Winter GD (1962). Formation of the scab and the rate of epithelization of superficial wounds in the skin of the young domestic pig. Nature.

[45] Brennan MJ, Kilbride BF, Wilker JJ, Liu JC (2017). A bioinspired elastin-based protein for a cytocompatible underwater adhesive. Biomaterials.

[46] Chang EI (2011). Vascular anastomosis using controlled phase transitions in poloxamer gels. Nat. Med..

[47] Wei SM, Pei MY, Pan WL, Thissen H, Tsai SW (2020). Gelatin hydrogels reinforced by absorbable nanoparticles and fibrils cured in situ by visible light for tissue adhesive applications. Polymers (Basel)..

[48] Yang KH (2021). Effect of photoinitiator on precursory stability and curing depth of Thiol-Ene Clickable gelatin. Polymers (Basel)..

[49] Lim KS (2019). Visible light cross-linking of gelatin hydrogels offers an enhanced cell microenvironment with improved light penetration depth. Macromol. Biosci..

[50] Sun M (2018). Synthesis and properties of gelatin methacryloyl (GelMA) hydrogels and their recent applications in load-bearing tissue. Polymers (Basel)..

[51] Yue K (2015). Synthesis, properties, and biomedical applications of gelatin methacryloyl (GelMA) hydrogels. Biomaterials.

[52] Arul V, Kartha R, Jayakumar R (2007). A therapeutic approach for diabetic wound healing using biotinylated GHK incorporated collagen matrices. Life Sci..

[53] Barrientos S, Stojadinovic O, Golinko MS, Brem H, Tomic-Canic M (2008). Growth factors and cytokines in wound healing. Wound Repair Regen..

[54] Kreuger J, Salmivirta M, Sturiale L, Giménez-Gallego G, Lindahl U (2001). Sequence analysis of heparan sulfate epitopes with graded affinities for fibroblast growth factors 1 and 2. J. Biol. Chem..

[55] Yang Y (2016). Tissue-integratable and biocompatible photogelation by the imine crosslinking reaction. Adv. Mater..

[56] Zhao X (2016). Photocrosslinkable gelatin hydrogel for epidermal tissue engineering. Adv. Healthc. Mater..

[57] Azuma K (2015). Biological adhesive based on carboxymethyl chitin derivatives and chitin nanofibers. Biomaterials.

[58] Galiano RD, Michaels JT, Dobryansky M, Levine JP, Gurtner GC (2004). Quantitative and reproducible murine model of excisional wound healing. Wound Repair Regen..

[59] Fukui T (2017). Liposome-encapsulated hemoglobin accelerates skin wound healing in diabetic dB/dB mice. Artif. Organs.

[60] McFarlane RM, Deyoung G, Henry RA (1965). The design of a pedicle flap in the rat to study necrosis and its prevention. Plast. Reconstr. Surg..

